# Supplementary figures and images for: Comparative Study of the Protective and Neurotrophic Effects of Neuronal and Glial Progenitor Cells-Derived Conditioned Media in a Model of Glutamate Toxicity In Vitro
Source: Biomolecules. 2023 Dec 13;13(12):1784. doi: 10.3390/biom13121784 (PMC10741670; doi:10.3390/biom13121784)

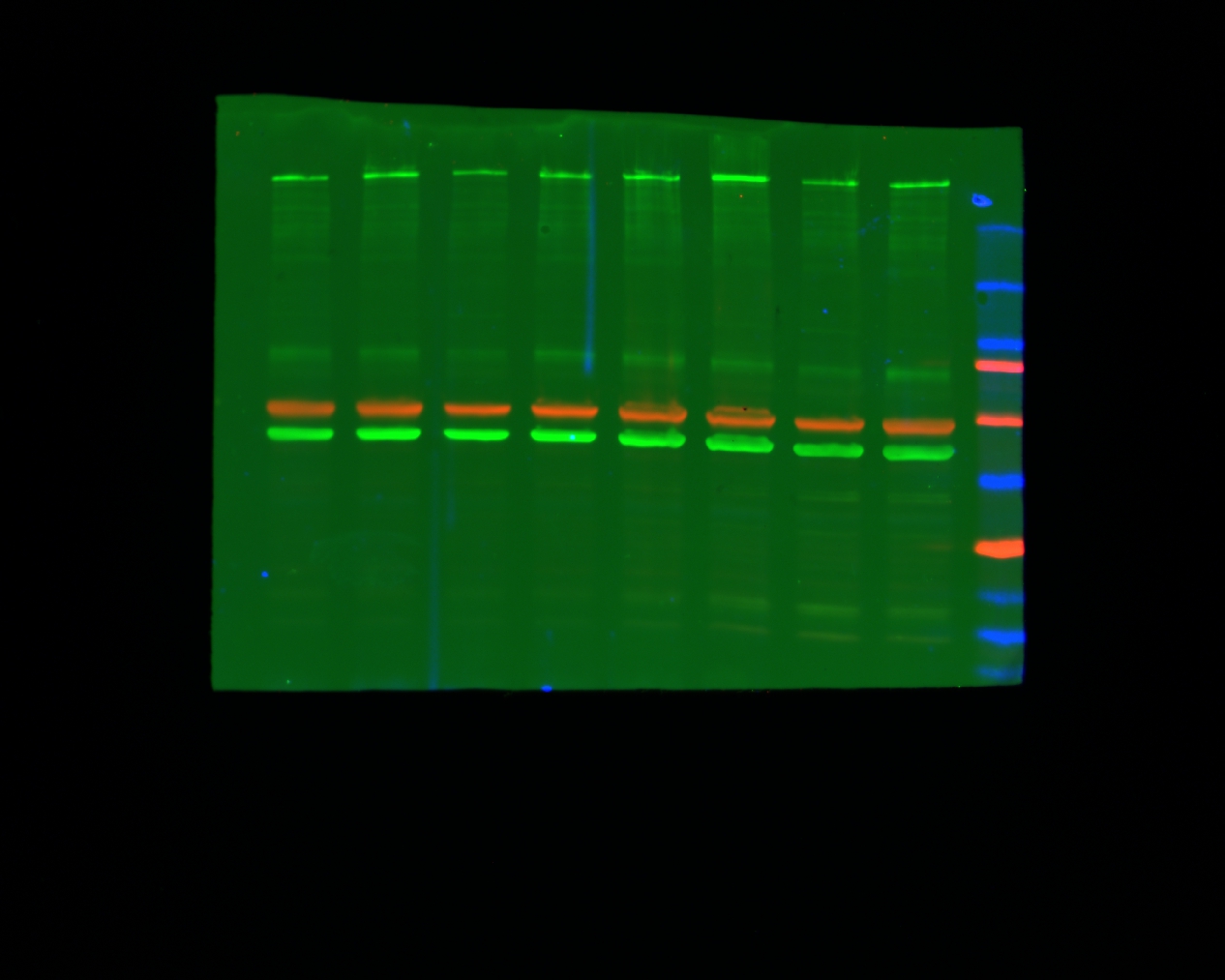

Supplement: Supplementary file 1 [file biomolecules-13-01784-s001.zip › GPC-CM_NGF.jpg]

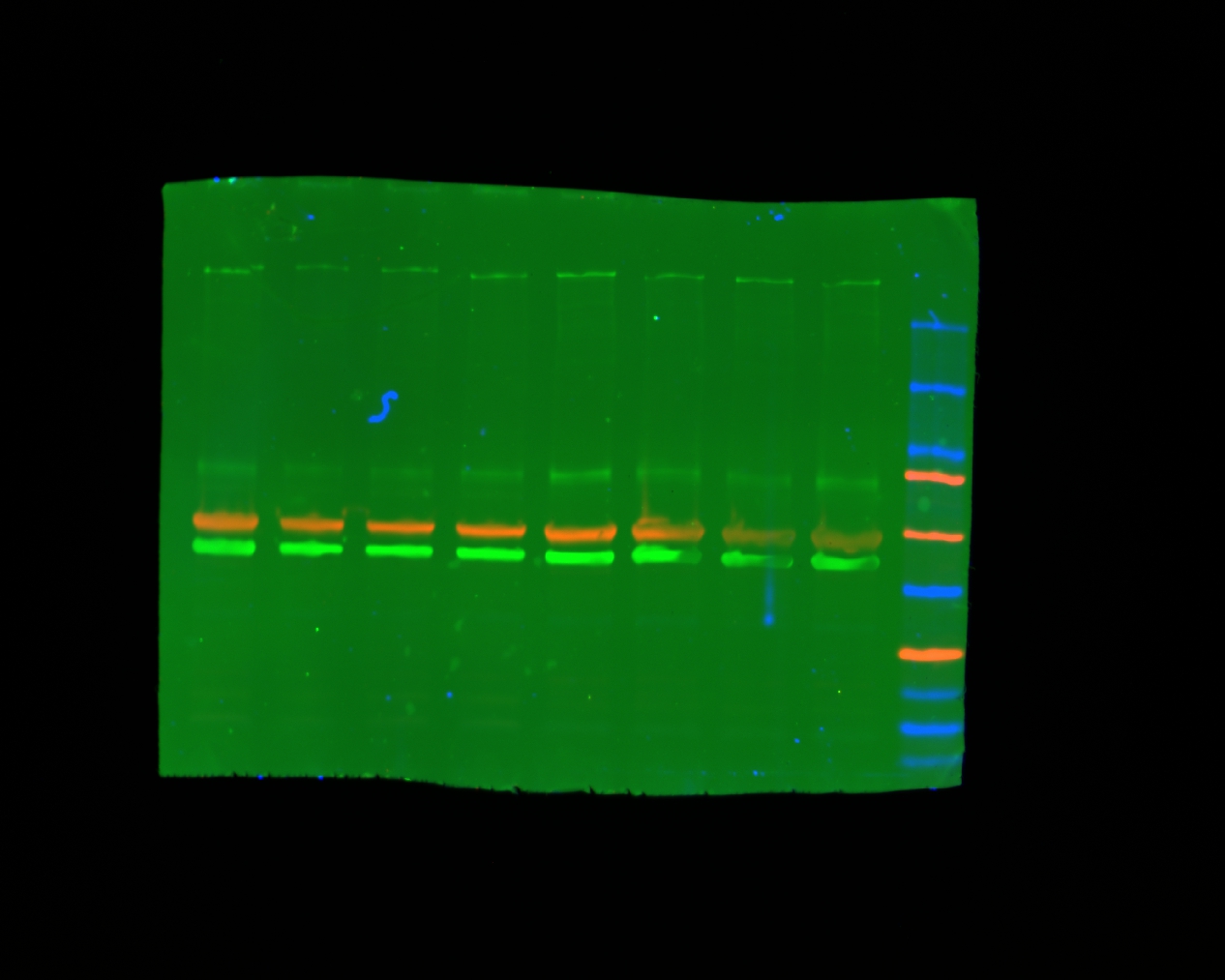

Supplement: Supplementary file 1 [file biomolecules-13-01784-s001.zip › NPC-CM_Control.jpg]
